# ASO Author Reflections: Use of Peritoneal Cancer Index (PCI) to Evaluate Carcinomatosis in Ovarian Cancer

**DOI:** 10.1245/s10434-020-08664-7

**Published:** 2020-05-28

**Authors:** Karin Stålberg, Björg Jónsdóttir

**Affiliations:** Department of Women’s and Children’s Health, Uppsala University, Uppsala, Sweden

## Past

The peritoneal cancer index (PCI) was introduced by Paul Sugarbaker to describe peritoneal carcinomatosis, initially for colorectal cancer and mesothelioma.^1^ PCI illustrates the distribution and the volume of carcinomatosis. In colorectal cancer, PCI shows a linear relationship with overall survival^2^ and affects postoperative quality of life.^3^ It is not generally recommended to operate on patients with colorectal carcinomatosis with PCI over 20.^4^ Previous studies on PCI in ovarian cancer are few and heterogeneous, suggesting cutoffs for surgery from 10 to 25, and since many of the studies were conducted, surgery has become more extensive.^5^,^6^

However, in most studies (both prospective and retrospective) on ovarian cancer, the FIGO stage is used to categorize the spread of the disease. The FIGO stage is quite a blunt tool with the majority of cases in stage IIIC, including patients with carcinomatosis ranging from a single peritoneal implant ≥ 2 cm in the upper abdomen to massive carcinomatosis. The assessment of PCI gives a more specific measurement and can be used both in preoperative imaging^7^ for deciding primary treatment (surgery or neoadjuvant chemotherapy) and in studies to compare populations.

## Present

The main aim of the present study^8^ was to find a PCI cutoff value for incomplete cytoreductive surgery (CRS) in patients with ovarian or fallopian tube cancer. Secondary aims were to identify reasons for open–close surgery and to compare surgical complications in relation to tumor burden. In the study cohort of 167 women, we can demonstrate that perioperative PCI was an excellent predictor of incomplete CRS and surgical complications. We identified two cutoff values for PCI: in patients with PCI > 24, the frequency of incomplete CRS was 32.7%, and 27% had a major complication; the corresponding figures for PCI > 33 were 71.4% incomplete CRS and 43% major complications. In 14 patients (total PCI 27–37), the carcinomatosis was considered inoperable and the abdomen was closed; the reason was massive infiltration on the small bowel in all cases.

## Future

Based on our results^8^ and literature, we suggest that neoadjuvant chemotherapy should be considered when PCI > 24, especially in fragile patients, to achieve optimal surgical outcome and minimize major complications. We recommend that PCI should be used as a standard parameter in clinical management of advanced gynecologic cancers and included in registers and research. Our findings support further studies on PCI in preoperative imaging as more accurate knowledge of tumor load could enhance patient selection before surgery. Our findings strengthen that the focus on preoperative radiology might be evaluation of total tumor burden, since this is strongly associated with operability.
